# Laminar Organization of FM Direction Selectivity in the Primary Auditory Cortex of the Free-Tailed Bat

**DOI:** 10.3389/fncir.2019.00076

**Published:** 2019-11-27

**Authors:** Silvio Macias, Kushal Bakshi, Michael Smotherman

**Affiliations:** Department of Biology, Texas A&M University, College Station, TX, United States

**Keywords:** auditory cortex, FM direction selectivity, current source density, echolocation, bats

## Abstract

We studied the columnar and layer-specific response properties of neurons in the primary auditory cortex (A1) of six (four females, two males) anesthetized free-tailed bats, *Tadarida* *brasiliensis*, in response to pure tones and down and upward frequency modulated (FM; 50 kHz bandwidth) sweeps. In addition, we calculated current source density (CSD) to test whether lateral intracortical projections facilitate neuronal activation in response to FM echoes containing spectrally distant frequencies from the excitatory frequency response area (FRA). Auditory responses to a set of stimuli changing in frequency and level were recorded along 64 penetrations in the left A1 of six free-tailed bats. FRA shapes were consistent across the cortical depth within a column and there were no obvious differences in tuning properties. Generally, response latencies were shorter (<10 ms) for cortical depths between 500 and 600 μm, which might correspond to thalamocortical input layers IIIb–IV. Most units showed a stronger response to downward FM sweeps, and direction selectivity did not vary across cortical depth. CSD profiles calculated in response to the CF showed a current sink located at depths between 500 and 600 μm. Frequencies lower than the frequency range eliciting a spike response failed to evoke any visible current sink. Frequencies higher than the frequency range producing a spike response evoked layer IV sinks at longer latencies that increased with spectral distance. These data support the hypothesis that a progressive downward relay of spectral information spreads along the tonotopic axis of A1 *via* lateral connections, contributing to the neural processing of FM down sweeps used in biosonar.

## Introduction

Numerous physiological studies of the auditory cortex in bats have revealed remarkable specializations for processing species-specific echolocation signals as well as a high diversity of the size and arrangement of functional fields (Suga, 1984, 2012; Dear et al., 1993; O’Neill, 1995; Hoffmann et al., 2008; Kössl et al., 2014, 2015). Some of the complex response properties observed in bats are believed to be derived from local intracortical circuits, but knowledge of the laminar differences in functional processing in bats is elusive. Layer-specific features of the bat auditory cortex have been reported recently in terms of neural oscillations (García-Rosales et al., 2019). As seen in other animals, it is presumed that neuronal response properties might vary systematically within the bat auditory cortical column reflecting discrete roles played by different layers for extracting key stimulus features *via* intracolumnar computational connections. In the neocortex, layer-dependent variations in minimum response latencies, tuning sharpness, and threshold have been described in A1 other mammals (Phillips and Irvine, 1981; Mendelson et al., 1997; Sugimoto et al., 1997; Wallace and Palmer, 2008; Atencio and Schreiner, 2010). However, depth-dependent variations in responses of the primary auditory cortex (A1) have not been reported in echolocating bats. Identifying any such patterns is an important step towards reconstructing the neural circuits (Atencio and Schreiner, 2010) bats rely upon to interpret their biosonar echoes.

The current study sought to determine the differences, if any, in the response properties of cells from different laminae in the A1 of the free-tailed bat *Tadarida* *brasiliensis*. The biosonar behavior of the free-tailed bat is typical of insectivorous bats that use multi-harmonic downward frequency modulated (FM) sweeps. In open space, free-tailed bat pulses are characterized as long (10–15 ms) narrow bandwidth FM chirps sweeping downward from around 25–20 kHz, but during target approach their pulse durations shorten while bandwidth increases dramatically *via* an elevation of the beginning frequency (Simmons et al., 1978; Schwartz et al., 2007). Extensive physiological details are already known about the free-tailed bat’s ascending auditory pathways (Pollak et al., 2011) but so far nothing is known about the functional organization of their auditory cortex. In a preliminary survey, we found that the majority of neurons in the free-tailed bat A1 were preferentially sensitive to downward FM sweeps used in their biosonar, and FM sweep selectivity in the A1 is known to arise from local intracortical networks (Razak and Fuzessery, 2006), so we decided to exploit this property of the system to closely examine the laminar characteristics of selectivity to the direction of frequency modulated (FM) sweeps.

FM direction selectivity arises through asymmetric inhibitory sidebands on the frequency response areas (FRAs; Suga, 1965a,b; Heil et al., 1992a,b; Fuzessery and Hall, 1996; Gordon and O’Neill, 1998; Razak and Fuzessery, 2002, 2006, 2008; Zhang et al., 2003). In those cases, lateral inhibition from frequencies below a neuron’s FRA can blunt responses to anything but downward FM sweeps. Both neighboring and spectrally distant frequencies outside of the FRA mediate neuronal response properties *via* horizontal inter-columnar projections (Kaur et al., 2004, 2005; Happel et al., 2010), and while most prior studies have focused on asymmetric inhibitory interconnections, there is also evidence of excitatory summation mechanisms shaping FM sweep directional sensitivity (Heil et al., 1992b) that are difficult to segregate from thalamocortical inputs but which might be contributing to the emergence of complex response properties in A1.

Using current source density (CSD) analysis, we tested the hypothesis that characteristic frequency and spectrally-distant non-characteristic frequency stimuli preferentially activate thalamocortical and horizontal pathways, respectively. The CSD analysis relies on the second spatial derivative of the local field potentials (LFPs) along the radial depth to localize synaptic inputs. The precise spatial and temporal information about the functional weights of synaptic activity (sinks) provided by this method point to the mechanism of their generation. It allows the tracing of neuronal information flow within each cortical column (Nicholson and Freeman, 1975; Mitzdorf, 1985; Kaur et al., 2004, 2005; Szymanski et al., 2009; Happel et al., 2010; Schaefer et al., 2015). Current sinks are an indicator of net excitatory synaptic current in a small volume of cortex surrounding the recording site. Current sources, in contrast, reflect passive return currents or hyperpolarizing activity (Mitzdorf, 1985). CSD enabled us to compare mean synaptic activity in response to different pure tones of changing frequency and FM sweeps of different directions.

## Materials and Methods

### Surgical Preparation

Animals were group-housed in an artificial habitat at Texas A&M University (TAMU) with a reversed light cycle. Recordings were made in six bats (four females, two males) weighing 12–14 g. Bats were anesthetized with a solution of sodium pentobarbital (80 mg/kg, Euthasol, Virbac AH, Inc., Fort Worth, TX, USA). Although the use of sodium pentobarbital reduces the response bandwidth and sensitivity of cortical neurons, there are no obvious layer-specific differences in the effects of anesthesia (Gaese and Ostwald, 2001). The skin and temporal muscles were cut and removed, and a post was attached to the skull at the midline using cyanoacrylate gel. A craniotomy (~2 × 2 mm) was made using a scalpel blade to expose the left auditory cortex. Blood vessels around the medial cerebral artery were very consistent from bat to bat. This allowed us to locate the A1 and use the vessels as reference points for stereotaxic measurements. Individually anesthetized bats were placed in a body mold made of plastic foam, and the head was tightly fixed by a rod attached to a metal holder. All experiments were carried out according to the National Institutes of Health guidelines and were approved by the TAMU Institutional Animal Care and Use Committee.

### Stimulation and Recording

Acoustic signals were digitally synthesized and controlled using a custom-written program in Matlab (R2018a, MathWorks, Natick, MA, USA). Stimuli were generated at a sampling rate of 250 kHz with a National Instruments card (NI USB-6356). The audio signal was transferred to an audio amplifier (SONY, STR-DE197). The acoustic stimuli were broadcast to the bat with a calibrated speaker (DaytonAudio, PTMini-6) located 10 cm from the bat’s ear. The calibration curve was obtained with a Brüel and Kjaer sound recording system (1/4-inch Microphone 4135, Microphone Preamplifier 2670, Brüel and Kjaer, Naerum, Denmark) connected to a conditioning microphone amplifier (Nexus 2690, Brüel and Kjaer, Naerum, Denmark). We presented the animal with a randomized series of pure tones (10 ms duration, 0.5 ms rise/fall time) at different sound pressure levels (step size: 10 dB, range: 20–80 dB SPL) and frequencies (step size: 5 kHz, range: 15–70 kHz). Each frequency-level combination was presented 20 times at an interval of 300 ms. In three of the six bats, we stimulated with downward and upward FM sweeps of various durations (1, 2, 3, 5, 10, 25 and 50 ms) and a constant bandwidth of 50 kHz (70–20 kHz). All FM sweeps were produced with a RMS level of 80 dB SPL.

Recordings were performed in a custom-built sound-attenuating room. Neuronal recordings were performed using probes from Cambridge NeuroTech (16 × 2 probe with 250 μm between shanks with sharpened tips and 50 μm spacing between recording sites along each shank, Cambridge NeuroTech, Cambridge, UK). Each shank had a thickness of 15 μm. Using a micromanipulator system (MX7600R, Siskiyou Corporation, Grants Pass, OR, USA), probes were inserted slowly into the brain, through the intact dura mater, and placed perpendicular to the pial surface. The tip of the recording probe was usually inserted down to depths of ~1,000 μm, which with a probe of 16 recording sites with a sampling interval of 50 μm placed the most superficial recording channels 250 μm below the cortical surface. This configuration allowed us to record from layers II to VI. Neuronal data were acquired with an OmniPlex D Neural Data Acquisition System (Plexon Inc., Dallas, TX, USA) at a sampling rate of 40 kHz (per channel) and 16-bit precision. A TTL pulse output from the National Instruments card was recorded on an analog channel of the Plexon data acquisition system to achieve synchronization between the neural recordings and acoustic stimulus broadcasts.

### Analysis of Neural Recordings

The raw signal was digitally bandpass-filtered offline (elliptic, 2nd order) between 500 and 3,000 Hz to obtain the multiunit activity. Multiunit activity was sorted into single-unit activity following the K-means clustering method using the Offline sorter application software (version 4.4.2, Plexon Inc., Dallas, TX, USA). We used a threshold of four times the standard deviation of the baseline noise for spike detection. Channels where the signal to noise ratio was lower than 12 dB were excluded from the data. From the raster plots, representing the spike-time vs. the trial number, we measured the number of spikes in a window of 50 ms after the stimulus onset for each frequency-level combination to build the FRAs. FRAs of each neuron were visualized as filled contour plots (contourc function, Matlab). From these, we calculated threshold curves as 25% of the maximum response. For each response, we calculated the minimum threshold (MT), characteristic frequency (CF; i.e., the frequency and level at the lowest tip of the threshold curve) and the bandwidth of the tuning curve 10 dB above MT. CF and bandwidth were used to calculate the *Q*_10_ (*Q*_10_ = CF/bandwidth) as an indicator of tuning sharpness. PSTHs (2 ms bin width) were produced for the response at the CF and 20 dB above MT. From these PSTHs, it was possible to measure the response latency, as the time at which the response reached 25% of the histogram peak, and the response duration, as the difference between response latency and the time when the response fell to 25% of the peak.

To quantify selectivity in response to FM sweeps with different directions, the direction selectivity index (DSI) was used (O’Neill and Brimijoin, 2002; Razak and Fuzessery, 2002, 2006, 2008; Morrison et al., 2018). The formula used was:

DSI=(D−U)/(D+U)

D and U are the maximum response magnitudes for downward and upward FM sweeps, respectively. While the DSI of each neuron was calculated using upward and downward sweeps of the same bandwidth, it was not necessarily calculated at the same sweep rate for the two directions because the maximum responses could occur at different sweep durations for the two sweep directions. Values of DSI range between −1 and +1, with more positive values indicating higher selectivity for the downward direction. DSI values greater than 0.6 indicate that the maximum response to the upward sweep was ≥75% lower than the maximum response to the downward sweep.

We calculated the LFPs by bandpass-filtering the raw signal between 0.1 and 500 Hz and applied a notch filter at 60 Hz to remove power-line noise. Based on the LFP recordings across cortical layers, we calculated the one-dimensional CSD profile from the second spatial derivative of the LFP (Nicholson and Freeman, 1975; Mitzdorf, 1985, 1986; Steinschneider et al., 1992; Happel et al., 2010):

$$-\text{CSD}\approx \frac{\delta^{2}\phi (z)}{\delta z^{2}}=\frac{\phi (z+n\text{Δ}z)-2\phi (z)+\phi (z-n\text{Δ}z)}{{\left(n\text{Δ}z\right)}^{2}}$$

where *Φ* is the field potential, *z* is the spatial coordinate perpendicular to the cortical laminae, Δ*z* is the sampling interval (50 μm), and n is the differentiation grid (Kaur et al., 2005; Happel et al., 2010). CSD calculation was performed using a modified version of the iCSDplotter toolbox (Pettersen et al., 2006). The estimations of the CSD at top and bottom channels were performed by previously described methods (Vaknin et al., 1988; Schaefer et al., 2015). Current sinks are interpreted to indicate depolarizing events such as active excitatory synaptic populations and axonal depolarization. Current sources, in contrast, reflect passive return currents (Mitzdorf, 1985) or hyperpolarizing events. We calculated the contours, using Matlab’s contour function, around the CSD sinks which surpassed 8% of the maximum sink amplitude of the CSD profile of the respective column. In these contours, we quantified the onset latency, measured as the minimum time of the contour, the peak, the duration, measured as the difference between the maximum and minimum times of the contour, and their vertical extent, measured as the difference between the maximum and minimum cortical depth of the contour.

### Immunohistochemistry and Cell Counts

A lethal dose of sodium pentobarbital was administered and the brain was dissected rapidly and fixed in 4% paraformaldehyde (0.1 M PBS, pH 7.4) for 24 h and transferred to 20% sucrose for an additional 18 h at 4°C. The brain was then embedded and 40 μm coronal sections were mounted to slides and allowed to dry overnight. Sections undergoing the Nissl staining procedure were rehydrated with distilled water and submerged in 1% cresyl violet solution for 5 min. For visualizing the inhibitory interneurons, primary antibodies marking parvalbumin (PV), somatostatin (SS), and calretinin (CR) were used to visualize each of the three different subpopulations of inhibitory interneurons. Nissl staining was used to visualize the overall cytoarchitecture of the brain. The primary auditory cortex was located using methods described previously (Martin del Campo et al., 2012).

Brightfield images (Olympus CX41, Olympus Scientific Solutions, Waltham, MA, USA; Lecia EC4 microscope camera, Leica Microsystems, Inc., Buffalo Grove, IL, USA) were imported into ImageJ (NIH, Bethesda, MD, USA) for analysis. The cortical depth was divided into 10 regions of 100 μm^2^ from the pia to the external capsule and we counted the number of cells and averaged them out of five sections in three bats.

## Results

### Laminar Characteristics of Neuronal Response Properties

**Figure 1:**
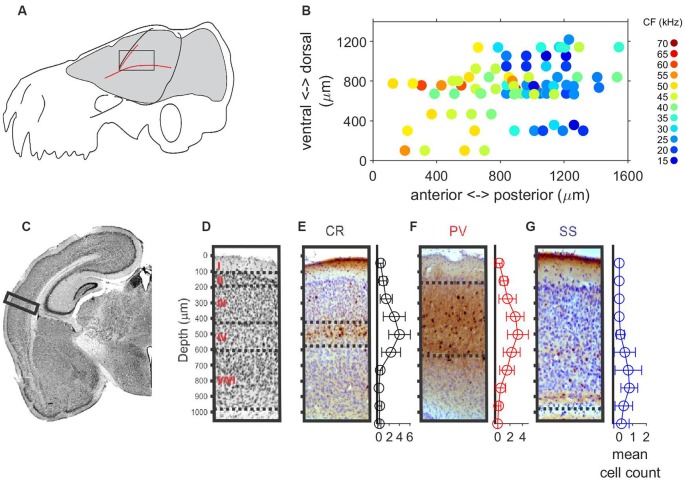
Localization, tonotopy and layer organization of the A1 penetrations in the free-tailed bat. **(A)** Schematic representation of the size and localization of the A1. **(B)** Topographical distribution of characteristic frequencies (CFs, color code) of neurons recorded at cortical depth between 500 mm and 600 mm. **(C)** Nissl-stained histological section in the Auditory cortex (AC). The rectangle marks the sampled region of AC. **(D)** Nissl-stained section with a horizontal line separating layers. **(E)** Coronal section stained for Calretinin (CR) and mean cell count of CR+ cells. **(F)** Coronal section stained for Parvalbumin (PV) and mean cell count of PV+ cells. **(G)** Coronal section stained for Somatostatin (SS) and mean cell count of SS+ cells.

Auditory responses were recorded along 64 penetrations in the left A1 of six anesthetized free-tailed bats (12, 11, 14, 12, 8 and 7 penetrations, respectively per bat), while presenting a set of stimuli changing in frequency and level. The location and size of the free-tailed bat’s A1 is schematically represented in Figure 1A. The courses of the blood vessels were used for localization of penetrations. Figure 1B shows the topographical distribution in the anterior-posterior and dorsal-ventral axes of units recorded at cortical depths between 500 μm and 600 μm. Units with lower CF were recorded in posterior regions of the A1 and an increase of the CF was observed in the anterior direction. The overall thickness of the intact A1 was around 1 mm (Figure 1C) and post-mortem fixation effects that caused the cortex to shrink in volume by approximately 15% were taken into account in the analyses. We used a combination of Nissl staining and antibody labeling to delineate borders of A1 laminae (Figures 1D–G). The low cell-body density of layer I was in sharp contrast with the high cell density of layer II in the Nissl-stained slides (Figure 1D). Borders between layers III–V could not easily be inferred from Nissl-stained cell counts, as noted previously (Anderson et al., 2009; Martin del Campo et al., 2012, 2014). When combined with antibody labeling of inhibitory interneurons, we were able to determine borders for layer III and IV. Borders of layer III were between depths with decreased cell density from layer II to the localization of calretinin-positive (CR) interneurons primarily in layer IV (Figure 1E). We observed parvalbumin-positive (PV) interneurons localized in layers II–V (Figure 1F), which is consistent with findings in A1 of other species (Martin del Campo et al., 2012, 2014). Somatostatin-positive (SS) interneurons localized primarily in deep layer VI (Figure 1G). There were no obvious cytoarchitecture markers separating layers V and VI.

**Figure 2:**
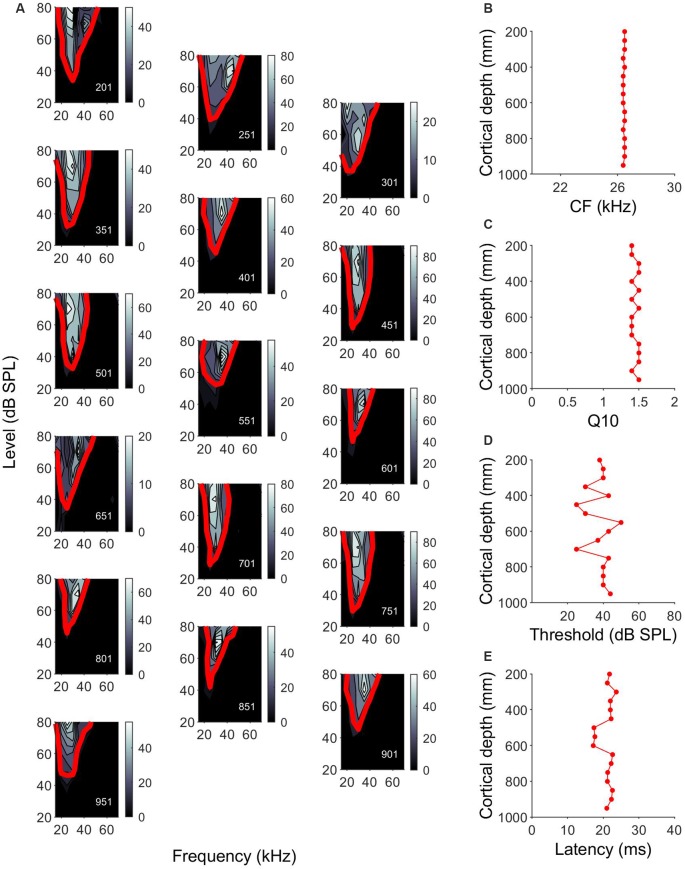
Cell response properties along a single penetration in the A1. **(A)** Single orthogonal penetration in A1 showing frequency response areas (FRAs) and tuning curves that have been arranged in a zigzag fashion. Color bar indicates the number of spikes. Red line indicates frequency tuning curves calculated 25% of the maximum number of spikes. Responses are units at depths of 201–951 μm (depth shown in the lower left corner of each panel). **(B–E)** Variation of CF, Q10, minimum threshold (MT) and response latency, respectively, across cortical depth for the example penetration shown in **(A)**.

As the same part of the auditory cortex was targeted in each experiment, using electrode holders oriented perpendicular to the surface, we were confident that all our penetrations had a similar orthogonal orientation. The units in the A1 had CFs of 15–70 kHz, with 50% of the units showing CFs between 20 kHz and 30 kHz. Plotting the FRAs and calculating the tuning curves allowed us to assess the characteristic frequency and tuning bandwidth of the units and assess the variability along cortical depth. Similar FRA shapes were often observed across the cortical depth. An example of changes of the frequency tuning curves in a single penetration is shown in Figure 2A. Figures 2B–E show the variation of characteristic frequency, tuning sharpness, MT and response latency as a function of the cortical depth. In this penetration, there was no variation in CF or *Q*_10_ values with increasing depth (Figures 2B,C). MT was very variable across depth, showing no particular pattern (Figure 2D). Latencies measured between 500 and 600 μm were around 5 ms shorter than those measured at other cortical depths (Figure 2E).

**Figure 3:**
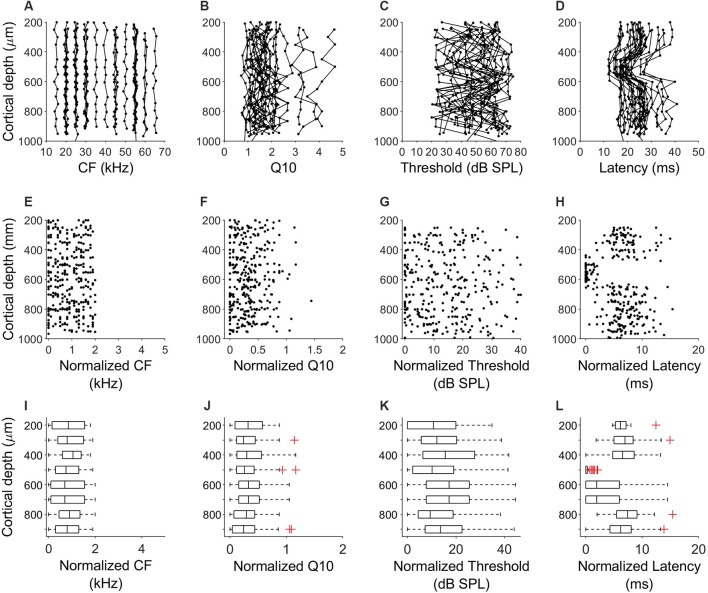
Laminar characteristics of neuronal response properties in the A1. **(A–D)** Variation of CF, Q10, MT and response latency across cortical depth for all the 64 penetrations in six bats. Each line represents a penetration. **(E–H)** Variation of the normalized values of CF, Q10, MT and response latency normalized to the minimum value of each column. **(I–L)** Comparison of the normalized values across depth. Data were grouped in ranges of 100 μm.

**Figure 4:**
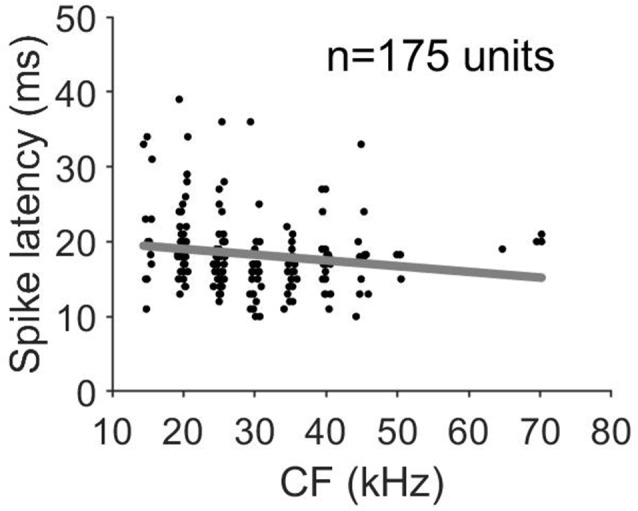
Response latency as a function of CF for units recorded at cortical depths between 500 and 600 μm.

In all penetrations, units had similar CF with a maximum difference of 2 kHz between the lowest and highest CF. Figure 3A shows the variation of CF of the 64 penetrations across cortical depth. We quantified the sharpness of tuning by calculating the Q10 values for 360 units from orthogonal penetrations. Q10 as a function of cortical depth for the 64 penetrations is represented in Figure 3B. Each line represents a column. We found a similar Q10 value across depth. MT showed a high variability but there were no obvious depth-specific trends (Figure 3C). Latency, on the other hand, showed an obvious pattern of variation across depth, with 5 ms shorter response latencies occurring at cortical depths between 500 and 600 μm in every penetration (64/64, Figure 3D). In order to quantify the variability and identify depth-specific trends, we normalized these parameters to the minimum value within each column and plotted the results as a function of cortical depth (Figures 3E–H). We did not find any depth-specific trends for CF (Figure 3E), Q10 (Figure 3F) or MT (Figure 3G). However, in the response latency, normalized values around zero were located at depths between 500 and 600 μm (Figure 3H). We compared the normalized values across depth by grouping the units in depth ranges of 100 μm (Figures 3I–L). There were no significant differences in normalized CF, *Q*_10_ and MT between depths (Figures 3H–J, Kruskal–Wallis One Way Analysis of Variance on Ranks, *H* = 3.27, 4.35 and 10.69, *p* = 0.8, 0.7 and 0.15, respectively). Normalized values for response latency were significantly different for depths between 500 and 600 μm (Figures 3H,L = 128.24, *p* < 0.001, Dunn’s test, *p* < 0.05). The relationship between CF and response latency for units recorded at cortical depths between 500 and 600 μm is shown in Figure 4. We found a weak negative correlation between CF and response latency (Pearson correlation, *R* = −0.153; *p* = 0.043), where longer latencies are found in units with lower CF.

### Laminar Characteristics of FM Direction Selectivity

**Figure 5:**
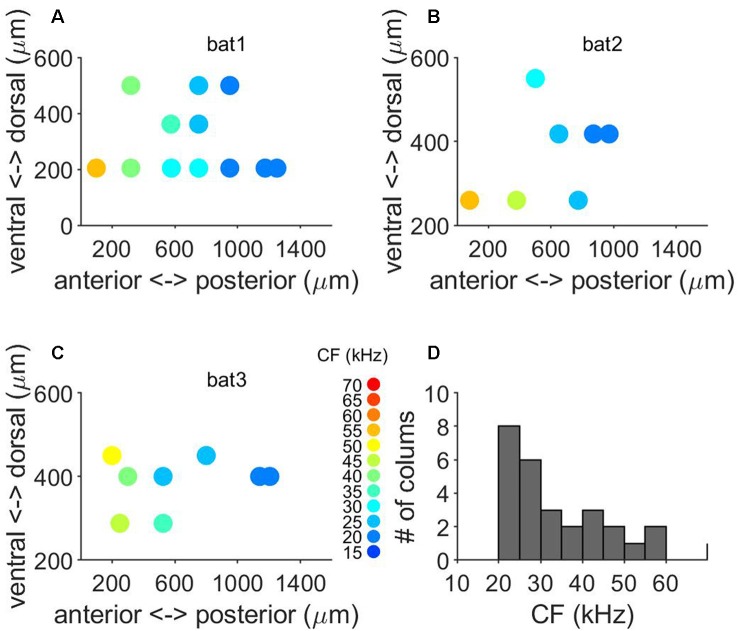
Tonotopy and frequency representation of frequency modulated (FM) direction selectivity. **(A–C)** Topographical distribution of CF in the three where we tested for FM direction selectivity. **(D)** Range of frequencies of columns tested for FM direction selectivity.

**Figure 6:**
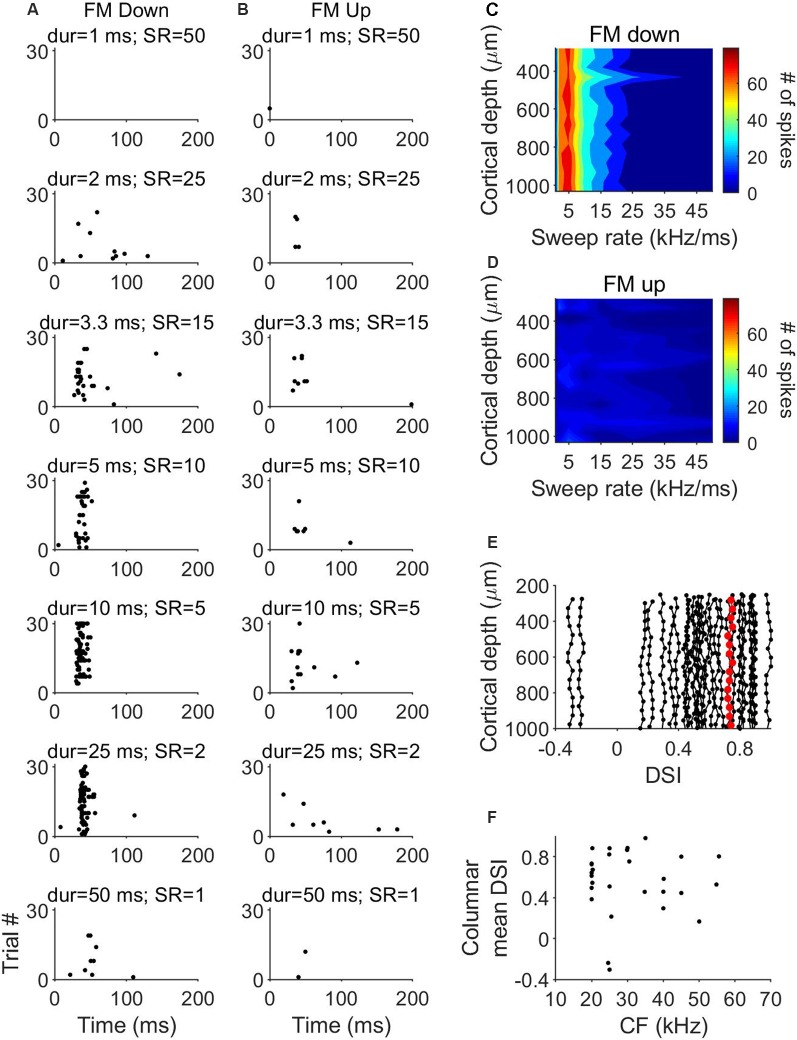
Laminar characteristics of FM direction-selectivity. **(A)** Dot raster plot of the response of a cortical neuron to downward FM sweeps of different sweep rates. **(B)** Dot raster plot of the response of the same cortical neuron to upward FM sweeps of different sweep rates. **(C)** Example laminar profile of the response to downward FM sweeps of different sweep rates. **(D)** Response of the same penetration shown in **(C)** to upward FM sweeps. **(E)** Distribution of direction selectivity index (DSI) as a function of cortical depth in 27 penetrations. Data highlighted in red correspond to penetration represented in **(C,D)**. **(F)** Columnar DSI as a function of the CF.

We determined the FM direction selectivity by recording the responses to FM sweeps with various durations (1–50 ms) but a constant bandwidth of 50 kHz. Direction selectivity was tested in 27 penetrations (373 units) across the tonotopically organized area of the A1 of three bats (Figures 5A–C). Columnar CFs of the studied units ranged from 15 to 65 kHz (Figure 5D). From these 373 units, 208 units showed a higher number of spikes for the downward FM sweep. A neuron was considered to be direction-selective for downward sweeps if the upward sweep elicited a response <25% of the response to the downward FM. An example unit is shown in Figures 6A,B. This cortical neuron showed a reliable response to downward FM sweeps with sweep rates between 2 and 15 kHz/ms (Figure 6A), whereas in response to upward FM sweeps it produced a very small number of spikes (Figure 6B). This unit showed a DSI of 0.72, indicating its preference for downward FM sweeps.

Overall, FM direction selectivity did not show variations across cortical depth. We plotted heat maps of the response magnitude as a function of sweep rate of the units in each penetration. An example column is shown in Figure 6C. The higher number of spikes is represented in red and the lowest number is represented in blue. When stimulated with downward FM sweeps, the 16 units recorded in this penetration showed a very similar pattern, with increased activity to sweep rates between 2 and 15 kHz/ms (Figure 6C). However, there was a very low number of spikes in each channel in response to the upward FM sweeps (Figure 6D). To quantify the possible differences in direction selectivity, we plotted the variation of the DSI across cortical depth for each of the 27 penetrations (Figure 6E). The maximum DSI difference between depths was 0.02. This indicates that there are no differences in direction selectivity between cortical layers. We did not find evidence of a relationship between the mean columnar CF and the mean columnar DSI (Figure 6F).

### Current Source Density Profiles

**Figure 7:**
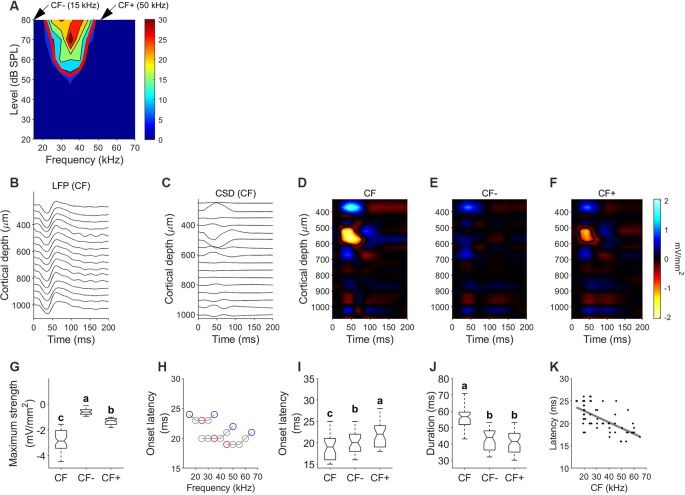
Current density profiles in response to pure tones. **(A)** Example FRA and tuning curve. **(B)** Local field potentials (LFPs) depth profile measured in response to the CF (35 kHz). **(C)** Current source densities (CSD) derived from the LFP profile shown in **(B)**. Time *0* corresponds to stimulus onset, negative values corresponding to current sinks. **(D)** CSD profile in response to characteristic frequency (CF = 35 kHz; 80 dB SPL). **(E)** CSD profile in response to a frequency lower than the FRA (CF−=15 kHz; 80 dB SPL). **(F)** CSD profile in response to a frequency higher than the FRA (CF+ = 50 kHz; 80 dB SPL). **(G)** Comparison of the maximum strength of the current sink measured in response to CF, CF− and CF+. **(H)** Variation of the onset latency of the current sink of three different columns with different CF. In each plot, black circles indicate CF−, red circles indicate CF and blue circles indicate CF+. **(I)** Comparison of onset latency. **(J)** Comparison of sink duration. In each comparison different letters represent significant differences after Kruskal–Wallis One Way Analysis of Variance on Ranks and Dunn’s test (*p* < 0.05). **(K)** Latency of initial current sink as a function of penetration’s CF. Gray line represents correlation line after Pearson correlation analysis.

The data shown in Figure 7 represent a typical example of a column tuned to frequencies between 20 and 45 kHz with CF of 35 kHz. The FRA and tuning curve for a unit recorded at a depth of 523 μm is depicted in Figure 7A. Figures 7B,C show the LFP depth profile and the derived CSDs calculated in response to the CF at 80 dB SPL, respectively. Because CSD depth profiles are easier to read if displayed as color maps, we plot them using a color scale that displays current sinks or depolarizing events with “hot” colors such as yellow and red, current sources or hyperpolarizing events with “cool” tones of blue and white, and zero net current is plotted in black. Sink contours were calculated at 8% of the maximum sink strength among the three conditions, obtained at the CSD profile calculated for the CF. In this example, CSD profiles were obtained for the CF (Figure 7D) and two frequencies outside the FRA, corresponding to frequencies 5 kHz lower and higher than the FRA borders at 80 dB SPL [CF− (15 kHz) and CF+ (50 kHz), Figures 7E,F, respectively]. In response to the CF, the earliest and strongest current sink is located at a depth between 502 ± 44.1 μm and 634.7 ± 42.8 μm. We calculated the onset latency of the current sinks by measuring the minimum time value of the sink contour. The earliest sink had an onset latency of 19.09 ± 4.32 ms and a vertical extent of 132.95 ± 49.7 μm. This early sink was observed in all penetrations with evident frequency tuning properties. In 47/64 CSD profiles, stimulating with a pure tone lower than the CF of the FRA (CF−) did not evoke any visible current sink. In the remaining 17 profiles a very weak current sink was observed. Frequencies higher than the CF of the FRA (CF+) evoked current sinks at depths between 517.72 ± 38.1 and 612.34 ± 32.7 μm with a latency of 20.97 ± 2.72 ms and a vertical extent of 95.98 ± 51.7 μm. Pure tones at CF evoked a significantly stronger sink than that of CF− and CF+ (Kruskal–Wallis One Way Analysis of Variance on Ranks, *H* = 130.75, *P* < 0.001; Dunn’s test, *P* < 0.001). Onset latency of the current sinks evoked by CF was significantly shorter than that at other frequencies (Figure 7G). Figure 7H represents the onset latencies measured in the early sink observed at different frequencies for three different penetrations with different CF. In each trace, red circle represents CF, black circle represents CF− and blue circle represents CF+. In these three examples, onset latencies at CF− (black circle) and CF+ (blue circle) were longer than that measured at CF (red circle). The population data also reflect this (Figure 7I). Onset latencies at CF are shorter than those at CF− and CF+ (*H* = 17.2, *p* = 0.017). Current sinks evoked by CF showed longer duration (Figure 7J, *H* = 83.985, *p* < 0.001) and longer vertical extent (*H* = 90.467, *p* < 0.001). There were no significant differences in duration and vertical extent between sinks evoked by CF− and CF+. We found a strong correlation between the characteristic frequency of the column and the latency of the current sink of the corresponding CSD profile (Figure 7K, Pearson correlation, *R* = −0.64, *p* < 0.001). Higher CFs showed current sinks with shorter latencies.

**Figure 8:**
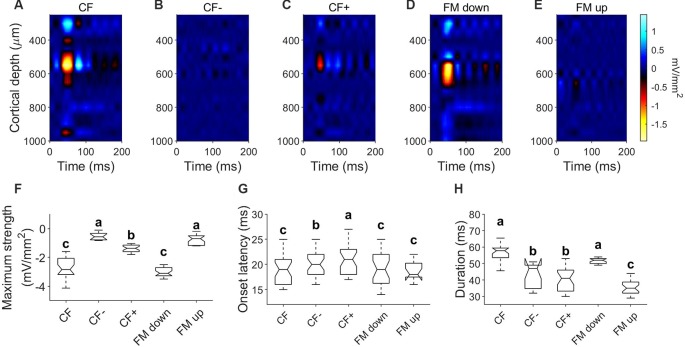
Current density profiles in response to FM sweeps. **(A–C)** CSD profiles in response to pure tones of CF (25 kHz; 80 dB SPL), CF− (15 kHz; 80 dB SPL) and CF+ (60 kHz; 80 dB SPL). **(D)** CSD profiles in response to downward FM sweep (sweep rate = 5 kHz/ms; 80 dB SPL). **(E)** CSD profiles in response to upward FM sweep (sweep rate = 5 kHz/ms; 80 dB SPL). **(F–H)** Comparison of parameters measured on the initial current sink of the CSD profiles in response to pure tones and FM sweeps. In each comparison, different letters represent significant differences after Kruskal–Wallis One Way Analysis of Variance on Ranks and Dunn’s test (*p* < 0.05).

We calculated CSD profiles in 27 penetrations in response to downward and upward FM sweeps of changing sweep rates at RMS level of 80 dB SPL. Since most of the units showed a preference for downward FM sweeps, CSD profiles were calculated for the response to the downward FM sweep rate evoking the largest response and compared to its corresponding upward sweep response. Example data is shown in Figure 8. When stimulated with pure tones at the column CF, a current sink (similar to those described above) is present (Figure 8A). This current sink is not present in response to pure tones with a CF− (Figure 8B) and occurred with a weaker strength relative to CF in response to a CF+ (Figure 8C). The CSD profile calculated in response to a downward FM sweep of 5 kHz/ms (Figure 8D) showed a current sink of 57.78 ± 8.11 ms long with a mean latency of 19.22 ± 3.4 ms, located between 517 ± 52.72 and 647.46 ± 32.9 μm. This sink was not present in 20/27 CSD profiles in response to upward FM sweeps (Figure 8E). There were no significant differences in the maximum strength, onset latency and duration between the sinks evoked by the CF and the FM down (Figures 8F–H, *p* > 0.05).

## Discussion

The organization of each layer within the A1 is complex, with different neuronal subpopulations, specific sources, targets, and local projection patterns, in addition to intra- and inter-laminar connections (Winer, 1992). Furthermore, the ascending and descending outputs from each layer target different regions of the auditory system, and likely have different purposes, adding to the complexity of the system (Winer, 2005). As a result, it is difficult to determine the general rules that capture how stimulus representations change between layers. Most receptive field properties exhibit systematic, depth-dependent changes including spectral bandwidth, sound intensity, frequency sweeps, spectral and temporal modulations, and vocalizations (Clarey et al., 1994; Mendelson et al., 1997; Sugimoto et al., 1997; Shen et al., 1999; Wallace and Palmer, 2008; Atencio and Schreiner, 2009). On the other hand, only preferred frequency (Abeles and Goldstein, 1970) and aurality (Brugge and Merzenich, 1973; Imig and Adrián, 1977; Middlebrooks et al., 1980; Clarey et al., 1994) have been shown to remain relatively constant.

In this study, we described the anatomical boundaries of cortical layers in the A1 of the free-tailed bat by examining differential cortical expression of three different calcium binding proteins and the presence of shorter response latencies and early current sinks. This combination has not been used before to delineate functional and neuroanatomical laminae borders in bat A1. In addition, we reported the systematic variation of frequency tuning properties and FM direction selectivity across cortical depth. FM sweeps are important and ubiquitous components of vocalizations. We show that the majority of the A1 of the free-tailed bat is selective for downward FM sweeps and point to the cortical circuits responsible for the recreation of this selectivity in the auditory cortex.

Our estimation of the border of the cortical layer coincided with that proposed for the mouse and the Pallid bat, *Antrozous* *pallidus* (Martin del Campo et al., 2012, 2014; Nguyen et al., 2017). PV cells in the mouse auditory cortex (AC) and the pallid bat A1 extends through layer V. These authors reported that that layer V was generally distinguishable from layer IV by having larger, less dense pyramidal cells than layer IV, and thus borders could be loosely estimated based on lower cell density estimates from Nissl stain measurements (Martin del Campo et al., 2012, 2014). Cortical depth of layer IV in the free-tailed bat matches the location of this layer in the mouse and the pallid bat, where it has been described to be at 50% of the cortical depth.

We found a consistent trend of shorter latencies in layer IV and longer latencies in superficial and deeper layers. There are evidences of shorter latencies in the deep cortical layers in gerbils and guinea pigs (Sugimoto et al., 1997; Wallace and Palmer, 2008). The results from the cat A1 report mixed findings. In one study, shorter latencies were observed in layers IV and V compared to the superficial layers (Phillips and Irvine, 1981). In a different study of the cat A1 (Mendelson et al., 1997), shorter response latencies to pure tones were recorded at the layer III/IV border than in layer V. However, since only two units were recorded in layer VI, the authors could not make conclusive statements about the response properties of this layer. We found a trend of shorter latencies at 500–600 μm, the putative thalamocortical input layer. This may mean that thalamic afferents can directly activate pyramidal cells in layer IV without any intrinsic relay from layers III or IV, as suggested in rats (Kimura et al., 2003) and cats (Huang and Winer, 2000). This is corroborated by the occurrence of a strong initial current sink in the CSD profiles calculated in response to the CF, which indicates feedforward input from afferent thalamocortical projections terminating in granular layers III/IV (Schroeder et al., 2003; Chen et al., 2007; Sakata and Harris, 2009; Atencio and Schreiner, 2010).

Mendelson et al. (1997) observed that response latencies of units in the auditory cortex of the cat appears to be weakly correlated with CF. A similar result to that described in this study for the spike response, where units with longer latencies showed lower CF, and for the current sink observed in layer IV. This negative correlation between response latency and CF has been described in the central nucleus of the inferior colliculus (Schreiner and Langner, 1988), implicating the traveling wave mechanism in determining the latency of response in the inferior colliculus and the A1 (Langner et al., 1987). Heil and Irvine (1997) analyzed the effect of amplitude, phase, and CF on first spike latency in both auditory nerve and A1 in cats. In both structures, latency decreases with increasing CF and in auditory cortex the latency decreases by almost 10 ms from 1 to 40 kHz, whereas latency differences in the auditory nerve are within 2–3 ms, so although the source is peripheral (Basilar membrane mechanics), the differences are exaggerated along the ascending auditory pathway.

Laminar differences in tuning bandwidth have been described in the A1 of cats, mice, gerbils and guinea pigs (Abeles and Goldstein, 1970; Wallace and Palmer, 2008; Atencio and Schreiner, 2009, 2010). In the A1 of the cat, there were disproportionately more broadly tuned neurons in the deep layers than in the superficial layers (Abeles and Goldstein, 1970; Atencio and Schreiner, 2009, 2010). A similar result was reported in the A1 of the guinea pig, where mean *Q*_10_ value for tuning in layers IV–VI was significantly smaller than for layers I–III (Wallace and Palmer, 2008). Evidence from studies in the gerbil show laminar differences with *Q*_10_ values higher in layers III/IV than in layers I or VI (Sugimoto et al., 1997). Oonishi and Katsuki (1965) suggested that some broadly tuned units may integrate multiple inputs from sharply tuned units in other layers and as well as horizontal intracortical connections which have been confirmed using CSD analysis. Kaur et al. (2005) showed that CF stimuli produced CSD profiles with prominent initial current sinks in layers III and IV, corresponding to the layers receiving lemniscal inputs from medial geniculate nucleus. In contrast, stimuli three octaves below characteristic frequency produced initial current sinks mainly in the infragranular layers. This study further supports the hypothesis that relay of thalamocortical information *via* horizontal intracortical projections may be the basis of broad spectral integration reflected in wider tuning in the deeper layers of the auditory cortex. Characteristic frequencies and tuning sharpness in the A1 of the free-tailed bat are constant across cortical layers. Although we spike sorted the multiunit recordings obtained from low impedance recording contacts, having the same neurons recorded in neighboring channels it is still a possibility. However, our data do not differ from findings in other bat species like *Pteronotus parnelli*, *Myotis lucifugus* or *Eptesicus fuscus* (Suga, 1965b, 1977; Suga and Jen, 1976; Suga and Manabe, 1982; Asanuma et al., 1983; Jen et al., 1989), where tuning properties are similar across cortical depth. This could be a common feature in echolocating bats. This does not imply that spectral integration is not taking place, involving information about CF and spectrally-distant non-CF stimuli. Based on our observation of shorter latencies in current sinks evoked in layer IV by frequencies higher than the FRA (CF+) compared to CF stimuli, we proposed that this spectral integration initially emerges from lemniscal thalamocortical inputs to layer IV, whereupon it is relayed along the tonotopic axis to cortical areas representing lower CFs by way of downward-biased horizontal pathways. This is based on our observation that higher CFs showed shorter latencies.

In the A1 of the free-tailed bat, selectivity to FM direction was strongly biased to respond preferentially to downward FM sweeps and this selectivity remains relatively constant across cortical depth. Early low-frequency inhibitory sidebands and asymmetrical facilitation shape selectivity for the downward sweep direction. The first prevents a response to upward sweeps, while the second is produced only in the downward sweep direction, suggesting that these may be discrete, complementary mechanisms (Razak and Fuzessery, 2006, 2008; Fuzessery et al., 2011). In most of the CSD profiles stimulating with a CF− pure tone there was no observable current sink and in those where a sink was present, the strength was very weak compared to that of the CF or CF+. The absence of sinks at CF− does not necessarily indicate the presence of inhibition but the absence of any excitatory connection between the site tuned to a lower frequency and the recording site. However, the presence of current sources even when sinks are missing could indicate that presence of inhibitory processes instead of return currents. On the other hand, in every site we observed a current sink evoked by CF+, with a shorter latency than that of the CF. This might be caused by the fact that the LFPs in the CF region are picking up excitatory synaptic currents that decrease spike threshold but are not strong enough to drive spiking. In addition, this could suggest the existence of a horizontal intracortical connection targeted on layer IV that evoked an excitation before the excitation by the CF. FM direction selectivity is present in the inferior colliculus and the medial geniculate body (O’Neill and Brimijoin, 2002; Razak and Fuzessery, 2006, 2008; Fuzessery et al., 2011). Thus, the possibility that cortical FM selectivity is inherited from afferent inputs is still plausible, although studies with GABA antagonists that suggest that the A1 recreates FM direction selectivity (Razak and Fuzessery, 2006). The data presented in this study support the mechanisms for implementation of direction selectivity in the A1 and bring insights about the wiring of the A1 to preferentially process downward FM sweeps.

## Data Availability Statement

The datasets generated for this study are available on request to the corresponding author.

## Ethics Statement

The animal study was reviewed and approved by Texas A&M University Institutional Animal Care and Use Committee.

## Author Contributions

SM, KB, and MS conceived and designed the study, and analyzed and interpreted the data. SM contributed to analytical tools. SM and KB acquired the data. SM, KB, and MS drafted the manuscript. All authors took full responsibility for the integrity of the data and accuracy of the analysis.

## Conflict of Interest

The authors declare that the research was conducted in the absence of any commercial or financial relationships that could be construed as a potential conflict of interest.

## References

[B1] Abeles M. Goldstein M. H. (1970). Functional architecture in cat primary auditory cortex: columnar organization and organization according to depth. J. Neurophysiol. 33, 172–187. 10.1152/jn.1970.33.1.172 5411512

[B2] Anderson L. Christianson G. Linden J. (2009). Mouse auditory cortex differs from visual and somatosensory cortices in the laminar distribution of cytochrome oxidase and acetylcholinesterase. Brain Res. 1252, 130–142. 10.1016/j.brainres.2008.11.037 19061871

[B3] Asanuma A. Wong D. Suga N. (1983). Frequency and amplitude representations in anterior primary auditory cortex of the mustached bat. J. Neurophysiol. 50, 1182–1196. 10.1152/jn.1983.50.5.1182 6644366

[B4] Atencio C. A. Schreiner C. E. (2009). Laminar diversity of dynamic sound processing in cat primary auditory cortex. J. Neurophysiol. 103, 192–205. 10.1152/jn.00624.2009 19864440 PMC2807218

[B5] Atencio C. A. Schreiner C. E. (2010). Columnar connectivity and laminar processing in cat primary auditory cortex. PLoS One 5:e9521. 10.1371/journal.pone.0009521 20209092 PMC2831079

[B6] Brugge J. F. Merzenich M. M. (1973). Responses of neurons in auditory cortex of the macaque monkey to monaural and binaural stimulation. J. Neurophysiol. 36, 1138–1158. 10.1152/jn.1973.36.6.1138 4761724

[B7] Chen C.-M. Lakatos P. Shah A. S. Mehta A. D. Givre S. J. Javitt D. C.. (2007). Functional anatomy and interaction of fast and slow visual pathways in macaque monkeys. Cereb. Cortex 17, 1561–1569. 10.1093/cercor/bhl067 16950866

[B8] Clarey J. C. Barone P. Imig T. J. (1994). Functional organization of sound direction and sound pressure level in primary auditory cortex of the cat. J. Neurophysiol. 72, 2383–2405. 10.1152/jn.1994.72.5.2383 7884466

[B9] Dear S. P. Fritz J. Haresign T. Ferragamo M. Simmons J. A. (1993). Tonotopic and functional organization in the auditory cortex of the big brown bat, Eptesicus fuscus. J. Neurophysiol. 70, 1988–2009. 10.1152/jn.1993.70.5.1988 8294966

[B12] Fuzessery Z. M. Hall J. C. (1996). Role of GABA in shaping frequency tuning and creating FM sweep selectivity in the inferior colliculus. J. Neurophysiol. 76, 1059–1073. 10.1152/jn.1996.76.2.1059 8871220

[B13] Fuzessery Z. M. Razak K. A. Williams A. J. (2011). Multiple mechanisms shape selectivity for FM sweep rate and direction in the pallid bat inferior colliculus and auditory cortex. J. Comp. Physiol. A 197, 615–623. 10.1007/s00359-010-0554-0 20596868 PMC3188416

[B14] Gaese B. H. Ostwald J. (2001). Anesthesia changes frequency tuning of neurons in the rat primary auditory cortex. J. Neurophysiol. 86, 1062–1066. 10.1152/jn.2001.86.2.1062 11495976

[B15] García-Rosales F. Röhrig D. Weineck K. Röhm M. Lin Y.-H. Cabral-Calderin Y.. (2019). Laminar specificity of oscillatory coherence in the auditory cortex. Brain Struct. Funct. 224, 2907–2924. 10.1007/s00429-019-01944-3 31456067

[B16] Gordon M. O’Neill W. E. (1998). Temporal processing across frequency channels by FM selective auditory neurons can account for FM rate selectivity. Hear. Res. 122, 97–108. 10.1016/s0378-5955(98)00087-2 9714578

[B17] Happel M. F. Jeschke M. Ohl F. W. (2010). Spectral integration in primary auditory cortex attributable to temporally precise convergence of thalamocortical and intracortical input. J. Neurosci. 30, 11114–11127. 10.1523/jneurosci.0689-10.2010 20720119 PMC6633479

[B18] Heil P. Irvine D. R. (1997). First-spike timing of auditory-nerve fibers and comparison with auditory cortex. J. Neurophysiol. 78, 2438–2454. 10.1152/jn.1997.78.5.2438 9356395

[B19] Heil P. Langner G. Scheich H. (1992a). Processing of frequency-modulated stimuli in the chick auditory cortex analogue: evidence for topographic representations and possible mechanisms of rate and directional sensitivity. J. Comp. Physiol. A 171, 583–600. 10.1007/bf00194107 1494138

[B20] Heil P. Rajan R. Irvine D. R. (1992b). Sensitivity of neurons in cat primary auditory cortex to tones and frequency-modulated stimuli. II: organization of response properties along the ‘isofrequency’dimension. Hear. Res. 63, 135–156. 10.1016/0378-5955(92)90081-w 1464567

[B21] Hoffmann S. Firzlaff U. Radtke-Schuller S. Schwellnus B. Schuller G. (2008). The auditory cortex of the bat Phyllostomus discolor: localization and organization of basic response properties. BMC Neurosci. 9:65. 10.1186/1471-2202-9-65 18625034 PMC2483289

[B22] Huang C. L. Winer J. A. (2000). Auditory thalamocortical projections in the cat: laminar and areal patterns of input. J. Comp. Neurol. 427, 302–331. 10.1002/1096-9861(20001113)427:2<302::aid-cne10>3.0.co;2-j 11054695

[B23] Imig T. Adrián H. (1977). Binaural columns in the primary field (A1) of cat auditory cortex. Brain Res. 138, 241–257. 10.1016/0006-8993(77)90743-0 589474

[B24] Jen P. H.-S. Sun X. Lin P. J. (1989). Frequency and space representation in the primary auditory cortex of the frequency modulating batEptesicus fuscus. J. Comp. Physiol. A 165, 1–14. 10.1007/bf00613794 2585357

[B25] Kaur S. Lazar R. Metherate R. (2004). Intracortical pathways determine breadth of subthreshold frequency receptive fields in primary auditory cortex. J. Neurophysiol. 91, 2551–2567. 10.1152/jn.01121.2003 14749307

[B26] Kaur S. Rose H. Lazar R. Liang K. Metherate R. (2005). Spectral integration in primary auditory cortex: laminar processing of afferent input, *in vivo* and *in vitro*. Neuroscience 134, 1033–1045. 10.1016/j.neuroscience.2005.04.052 15979241

[B27] Kimura A. Donishi T. Sakoda T. Hazama M. Tamai Y. (2003). Auditory thalamic nuclei projections to the temporal cortex in the rat. Neuroscience 117, 1003–1016. 10.1016/s0306-4522(02)00949-1 12654352

[B28] Kössl M. Hechavarria J. Voss C. Macias S. Mora E. Vater M. (2014). Neural maps for target range in the auditory cortex of echolocating bats. Curr. Opin. Neurobiol. 24, 68–75. 10.1016/j.conb.2013.08.016 24492081

[B29] Kössl M. Hechavarria J. Voss C. Schaefer M. Vater M. (2015). Bat auditory cortex—model for general mammalian auditory computation or special design solution for active time perception? Eur. J. Neurosci. 41, 518–532. 10.1111/ejn.12801 25728173

[B30] Langner G. Schreiner C. Merzenich M. (1987). Covariation of latency and temporal resolution in the inferior colliculus of the cat. Hear. Res. 31, 197–201. 10.1016/0378-5955(87)90127-4 3446676

[B10] Martin del Campo H. N. Measor K. R. Razak K. A. (2012). Parvalbumin immunoreactivity in the auditory cortex of a mouse model of presbycusis. Hear. Res. 294, 31–39. 10.1016/j.heares.2012.08.017 23010334

[B11] Martin del Campo H. N. Measor K. R. Razak K. A. (2014). Parvalbumin and calbindin expression in parallel thalamocortical pathways in a gleaning bat, Antrozous pallidus. J. Comp. Neurol. 522, 2431–2445. 10.1002/cne.23541 24435957 PMC3997625

[B31] Mendelson J. Schreiner C. Sutter M. (1997). Functional topography of cat primary auditory cortex: response latencies. J. Comp. Physiol. A 181, 615–633. 10.1007/s003590050145 9449822

[B32] Middlebrooks J. C. Dykes R. W. Merzenich M. M. (1980). Binaural response-specific bands in primary auditory cortex (AI) of the cat: topographical organization orthogonal to isofrequency contours. Brain Res. 181, 31–48. 10.1016/0006-8993(80)91257-3 7350963

[B33] Mitzdorf U. (1985). Current source-density method and application in cat cerebral cortex: investigation of evoked potentials and EEG phenomena. Physiol. Rev. 65, 37–100. 10.1152/physrev.1985.65.1.37 3880898

[B34] Mitzdorf U. (1986). The physiological causes of VEP: current source density analysis of electrically and visually evoked potentials. Front. Clin. Neurosci. 3, 141–154.

[B35] Morrison J. A. Valdizón-Rodríguez R. Goldreich D. Faure P. A. (2018). Tuning for rate and duration of frequency-modulated sweeps in the mammalian inferior colliculus. J. Neurophysiol. 120, 985–997. 10.1152/jn.00065.2018 29790835

[B36] Nguyen A. Khaleel H. M. Razak K. A. (2017). Effects of noise-induced hearing loss on parvalbumin and perineuronal net expression in the mouse primary auditory cortex. Hear. Res. 350, 82–90. 10.1016/j.heares.2017.04.015 28460252

[B37] Nicholson C. Freeman J. A. (1975). Theory of current source-density analysis and determination of conductivity tensor for anuran cerebellum. J. Neurophysiol. 38, 356–368. 10.1152/jn.1975.38.2.356 805215

[B38] O’Neill W. E. (1995). “The bat auditory cortex,” in Hearing by Bats, eds Popper A. N. Fay R. R. (Heidelberg: Springer), 416–480.

[B39] O’Neill W. E. Brimijoin W. O. (2002). Directional selectivity for FM sweeps in the suprageniculate nucleus of the mustached bat medial geniculate body. J. Neurophysiol. 88, 172–187. 10.1152/jn.00966.2001 12091543 PMC3904363

[B40] Oonishi S. Katsuki Y. (1965). Functional organization and integrative mechanism on the auditory cortex of the cat. Jpn. J. Physiol. 15, 342–365. 10.2170/jjphysiol.15.342

[B41] Pettersen K. H. Devor A. Ulbert I. Dale A. M. Einevoll G. T. (2006). Current-source density estimation based on inversion of electrostatic forward solution: effects of finite extent of neuronal activity and conductivity discontinuities. J. Neurosci. Methods 154, 116–133. 10.1016/j.jneumeth.2005.12.005 16436298

[B42] Phillips D. P. Irvine D. R. (1981). Responses of single neurons in physiologically defined primary auditory cortex (AI) of the cat: frequency tuning and responses to intensity. J. Neurophysiol. 45, 48–58. 10.1152/jn.1981.45.1.48 7205344

[B43] Pollak G. D. Xie R. Gittelman J. X. Andoni S. Li N. (2011). The dominance of inhibition in the inferior colliculus. Hear. Res. 274, 27–39. 10.1016/j.heares.2010.05.010 20685288 PMC3762690

[B44] Razak K. A. Fuzessery Z. M. (2002). Functional organization of the pallid bat auditory cortex: emphasis on binaural organization. J. Neurophysiol. 87, 72–86. 10.1152/jn.00226.2001 11784731

[B45] Razak K. A. Fuzessery Z. M. (2006). Neural mechanisms underlying selectivity for the rate and direction of frequency-modulated sweeps in the auditory cortex of the pallid bat. J. Neurophysiol. 96, 1303–1319. 10.1152/jn.00020.2006 16775213

[B46] Razak K. A. Fuzessery Z. M. (2008). Facilitatory mechanisms underlying selectivity for the direction and rate of frequency modulated sweeps in the auditory cortex. J. Neurosci. 28, 9806–9816. 10.1523/JNEUROSCI.1293-08.2008 18815265 PMC2567824

[B47] Sakata S. Harris K. D. (2009). Laminar structure of spontaneous and sensory-evoked population activity in auditory cortex. Neuron 64, 404–418. 10.1016/j.neuron.2009.09.020 19914188 PMC2778614

[B48] Schaefer M. K. Hechavarría J. C. Kössl M. (2015). Quantification of mid and late evoked sinks in laminar current source density profiles of columns in the primary auditory cortex. Front. Neural Circuits 9:52. 10.3389/fncir.2015.00052 26557058 PMC4617414

[B49] Schreiner C. E. Langner G. (1988). Periodicity coding in the inferior colliculus of the cat:. II. Topographical organization. J. Neurophysiol. 60, 1823–1840. 10.1152/jn.1988.60.6.1823 3236053

[B50] Schroeder C. E. Smiley J. Fu K. G. McGinnis T. O’Connell M. N. Hackett T. A. (2003). Anatomical mechanisms and functional implications of multisensory convergence in early cortical processing. Int. J. Psychophysiol. 50, 5–17. 10.1016/s0167-8760(03)00120-x 14511832

[B51] Schwartz C. Tressler J. Keller H. Vanzant M. Ezell S. Smotherman M. (2007). The tiny difference between foraging and communication buzzes uttered by the Mexican free-tailed bat, *Tadarida brasiliensis*. J. Comp. Physiol. A 193, 853–863. 10.1007/s00359-007-0237-7 17503051

[B52] Shen J.-X. Xu Z.-M. Yao Y.-D. (1999). Evidence for columnar organization in the auditory cortex of the mouse. Hear. Res. 137, 174–177. 10.1016/s0378-5955(99)00149-5 10545645

[B53] Simmons J. A. Lavender W. Lavender B. Childs J. Hulebak K. Rigden M. (1978). Echolocation by free-tailed bats (*Tadarida*). J. Comp. Physiol. 125, 291–299. 10.1007/BF00656863

[B54] Steinschneider M. Tenke C. E. Schroeder C. E. Javitt D. C. Simpson G. V. Arezzo J. C.. (1992). Cellular generators of the cortical auditory evoked potential initial component. Electroencephalogr. Clin. Neurophysiol. 84, 196–200. 10.1016/0168-5597(92)90026-8 1372236

[B55] Suga N. (1965a). Functional properties of auditory neurones in the cortex of echo-locating bats. J. Physiol. 181, 671–700. 10.1113/jphysiol.1965.sp007791 5881249 PMC1357677

[B56] Suga N. (1965b). Responses of cortical auditory neurones to frequency modulated sounds in echo-locating bats. Nature 206, 890–891. 10.1038/206890a0 5839840

[B57] Suga N. (1977). Amplitude spectrum representation in the Doppler-shifted-CF processing area of the auditory cortex of the mustache bat. Science 196, 64–67. 10.1126/science.190681 190681

[B58] Suga N. (1984). Neural mechanisms of complex-sound processing for echolocation. Trends Neurosci. 7, 20–27. 10.1016/s0166-2236(84)80183-6

[B59] Suga N. (2012). Tuning shifts of the auditory system by corticocortical and corticofugal projections and conditioning. Neurosci. Biobehav. Rev. 36, 969–988. 10.1016/j.neubiorev.2011.11.006 22155273 PMC3265669

[B60] Suga N. Jen P. (1976). Disproportionate tonotopic representation for processing CF−FM sonar signals in the mustache bat auditory cortex. Science 194, 542–544. 10.1126/science.973140 973140

[B61] Suga N. Manabe T. (1982). Neural basis of amplitude-spectrum representation in auditory cortex of the mustached bat. J. Neurophysiol. 47, 225–255. 10.1152/jn.1982.47.2.225 7062098

[B62] Sugimoto S. Sakurada M. Horikawa J. Taniguchi I. (1997). The columnar and layer-specific response properties of neurons in the primary auditory cortex of Mongolian gerbils. Hear. Res. 112, 175–185. 10.1016/s0378-5955(97)00119-6 9367240

[B63] Szymanski F. D. Garcia-Lazaro J. A. Schnupp J. W. (2009). Current source density profiles of stimulus-specific adaptation in rat auditory cortex. J. Neurophysiol. 102, 1483–1490. 10.1152/jn.00240.2009 19571199

[B64] Vaknin G. DiScenna P. Teyler T. (1988). A method for calculating current source density (CSD) analysis without resorting to recording sites outside the sampling volume. J. Neurosci. Methods 24, 131–135. 10.1016/0165-0270(88)90056-8 3405010

[B65] Wallace M. Palmer A. (2008). Laminar differences in the response properties of cells in the primary auditory cortex. Exp. Brain Res. 184, 179–191. 10.1007/s00221-007-1092-z 17828392

[B66] Winer J. A. (1992). “The functional architecture of the medial geniculate body and the primary auditory cortex,” in Springer Handbook of Auditory Research, Vol. 1, The Mammalian Auditory Pathway: Neuroanatomy, eds Webster D. B. Popper A. N. Fay R. R. (New York, NY: Springer-Verlag), 222–409.

[B67] Winer J. A. (2005). Decoding the auditory corticofugal systems. Hear. Res. 207, 1–9. 10.1016/j.heares.2005.06.007 16091301

[B68] Zhang L. I. Tan A. Y. Schreiner C. E. Merzenich M. M. (2003). Topography and synaptic shaping of direction selectivity in primary auditory cortex. Nature 424, 201–205. 10.1038/nature01796 12853959

